# Wood-Mimicking Bio-Based Biporous Polymeric Materials with Anisotropic Tubular Macropores

**DOI:** 10.3390/polym13162692

**Published:** 2021-08-12

**Authors:** Vierajitha Srikanthan, Olivier Pitois, Philippe Coussot, Benjamin Le Droumaguet, Daniel Grande

**Affiliations:** 1 Univ Paris Est Creteil, CNRS, ICMPE, UMR 7182, 2 Rue Henri Dunant, 94320 Thiais, France; srikanthan@icmpe.cnrs.fr; 2 Univ Gustave Eiffel, CNRS, Ecole des Ponts ParisTech, UMR 8205 Laboratoire Navier, 5 Boulevard Descartes, CEDEX 2, 77454 Marne-la-Vallée, France; olivier.pitois@univ-eiffel.fr (O.P.); philippe.coussot@univ-eiffel.fr (P.C.)

**Keywords:** bio-based monomers, vanillin, biporous polymers, anisotropic macropores, double porogen templating approach

## Abstract

Understanding physical phenomena related to fluid flow transport in plants and especially through wood is still a major challenge for the scientific community. To this end, we have focused our attention on the design of wood-mimicking polymeric architectures through a strategy based on the double porogen templating approach which relies on the use of two distinct types of porogens, namely aligned nylon threads and a porogenic solvent, to produce macro- and nanoporosity levels, respectively. A bio-based phenolic functional monomer, i.e., vanillin methacrylate, was employed to mimic either hard wood or soft wood. Upon free-radical polymerization with a crosslinking agent in the presence of both types of porogenic agents, followed by their removal, biporous materials with anistotropic tubular macropores surrounded by a nanoporous matrix were obtained. They were further fully characterized in terms of porosity and chemical composition via mercury intrusion porosimetry, scanning electron microscopy and X-ray microtomography. It was demonstrated that the two porosity levels could be independently tuned by varying structural parameters. Further, the possibility to chemically modify the pore surface and thus to vary the material surface properties was successfully demonstrated by reductive amination with model compounds via Raman spectroscopy and water contact angle measurements.

## 1. Introduction

Wood is a building material that has been used through the ages. This naturally occurring material is peculiar, due to its specific behavior regarding moisture and water flow transport. It is indeed well-known that wood can endure successive water uptake-drying cycles without damaging. This uncommon physical behavior has attracted a growing interest from the research community [1,2,3]. Understanding the physical features that underlie such water uptake-drying cycles would probably lead to the possibility to build up synthetic materials with identical physical behavior. Wood’s structure is essentially made of elongated hollow cellular units (vessels or tracheids) directed along the direction of the tree trunk, and along which the water can a priori move easily (at least between pits), surrounded by a solid structure able to absorb water. For hardwood, this structure also contains elongated enclosed fibers able to store some free water. Remarkably, it was recently shown that the ability of water to flow through the matrix plays a critical role on the processes of spontaneous imbibition or drying. The spontaneous imbibition in hardwoods is strongly damped by the water absorption in the matrix, which affects the wetting properties in the vessels [3,4]; the drying under convection can be maintained at a constant rate thanks to the water motion in the matrix [5].

The specific properties of wood are thus certainly due to the combination of a complex porous structure and the particular chemical composition of the matrix (cell walls). From a structural viewpoint, wood is composed of cellulose and hemicellulose, and mainly of lignin, a crosslinked biopolymer that is derived from structurally similar monomers having a common point, a phenolic building block. In this context, we have focused on the design of model polymeric materials that can mimic both the porous structure (elongated vessels in a porous matrix) and the chemical composition of wood. Recently, Epps’s research group reported on the preparation and subsequent polymerization of methacrylic monomers arising from bio-based compounds originating from wood chemical composition, namely syringol and guaiacol [6,7]. Such chemical compounds, having relatively similar phenolic structures, can be easily transformed into the corresponding methacrylate in a straightforward fashion. In this regard, phenolic monomers arising from bio-based guaiacol, syringol and even vanillin have been used as model building blocks. In the same time, it is well-known that wood develops a very particular porous structure in which different levels of porosity can be observed. It is also worth noticing that two types of porous structures may exist depending on the type of wood, i.e., either softwood or hardwood. Several porous polymer-based structures mimicking wood have been so far reported. They are notably based on temperature induced phase separation [8,9] or 3D printing [10]. The porous polymers obtained display in that case a single porosity level that is far from the observed morphology of wood that exhibits hierarchical biporous structure. In comparison, doubly porous structures often present very interesting intrinsic physical properties that are notably related to fluid flow and transport. Such properties permit to envision these biporous frameworks in various applications, including tissue engineering [11,12], soil mechanics and civil engineering [13,14,15].

Herein, we develop a strategy based on the double porogen templating approach for the preparation of bio-based polymeric materials that exhibit two porosity levels as wood-mimicking model systems. The originality of this work relies on the anisotropic doubly porous polymeric materials that can be obtained in one pot from this methodology. Thus, these materials should better mimic the complex structure and morphology of wood. The larger porosity level consists of aligned tubular macropores presenting a regular pattern in the material, while the lower porosity level is produced by phase separation in the presence of a porogenic solvent. Different parameters have been investigated regarding both porosity levels. The solvent nature and volume fraction in the polymerization feed have been varied so as to investigate their impact on the lower porosity level. The as-obtained biporous materials have been thoroughly characterized in terms of porosity and chemical composition. We have notably demonstrated an unprecedented anisotropic porous morphology in such biporous polymeric materials. The possibility to modify the pore surface of such innovative materials has also been investigated by reductive amination of available aldehyde moieties arising from vanillin methacrylate, thus demonstrating the potential of such materials as a versatile functionalization platform.

## 2. Materials and Methods

### 2.1. Materials

Vanillin (V, 99%), methacrylic anhydride (94%), 4-dimethylaminopyridine (DMAP, 99%), ethylene glycol dimethacrylate (EGDMA, 98%), 2,2-dimethoxy-2-phenylacetophenone (DMPA), sodium hydroxide (NaOH, 0.5 M and 1 M), *tert*-butanol (*t*-BuOH, 99%), dodecan-1-ol (DOH, 98%), toluene (≥99.5%) were purchased from Sigma Aldrich. Hydrochloric acid (HCl, 37%), dichloromethane (CH_2_Cl_2_, 99.8%), ethyl acetate (EtOAc, 99.8%), and cyclohexane (99.8%) were supplied by Carlo Erba Reagents. 2-hydroxyethyl methacrylate (HEMA, 97%) was obtained from Alfa Aesar and Acros Organics, respectively. Ethanol (EtOH, 99.98%), methanol (MeOH, 99.9%), and 2-propanol (*i*-PrOH, 99.8%) were purchased from VWR Chemicals. 1-octanol (≥99%) was obtained from Janssen Chimica. All reagents were used without any further purification, except AIBN that was recrystallized from diethyl ether prior to use. 18.2 MΩ deionized water was filtered through a Milli-Q Plus purification pack.

### 2.2. Synthesis of Vanillin Methacrylate

The synthesis of vanillin methacrylate (VMA) was performed following a procedure reported elsewhere [16]. Vanillin (25 g, 0.164 mol), a catalytic amount of DMAP (1.5 mol% with respect to vanillin), and dichloromethane (50 mL) were introduced into a three-necked round bottom flask. Then, methacrylic anhydride (0.197 mol, 1.2 equiv. with respect to vanillin) was dropwise added to the solution using a dropping funnel. The reaction was then allowed to stir under inert atmosphere for 5 days under reflux. The reaction mixture was then cooled to room temperature and diluted in 150 mL ethyl acetate. The organic phase was successively washed twice with a sodium bicarbonate saturated solution (150 mL, until there was no further release of gas), once with a 1 M NaOH solution (150 mL), with a 0.5 M NaOH solution (150 mL), with a 0.1 M hydrochloric acid (150 mL), and finally with deionized water (150 mL). The organic phase was then dried over MgSO_4_, filtered off, and concentrated under vacuum to afford the pure product as a white solid (95% yield). NMR ^1^H (CDCl_3_; 400 MHz): *δ* (ppm) 9.95 (s; 1H; -C*H*O); 7.49 (d; *J*=9.3 Hz; 2H; 2 *H_Ar_*); 7.24 (d; *J*=1 Hz; 1H; *H_Ar_*); 6.38 (d; *J*=1.4 Hz; 1H; -C=C*H*_2_); 5.80 (d; *J*=1.4 Hz; 1H; -C=C*H*_2_); 3.89 (s; 3H; -O-C*H_3_*); 2.07 (s; 3H; -C=C-C*H_3_*). NMR ^13^C (CDCl_3_; 100 MHz): *δ* (ppm) 191.24 (-*C*HO); 164.94 (-*C=*O); 152.27 (*C_Ar_*); 145.38 (*C_Ar_*); 135.33 (*C_Ar_*); 135.29 (=*C*-CH_3_); 128.10 (=*C*H_2_); 124.92 (*C_Ar_*); 123.62 (*C_Ar_*); 110.95 (*C_Ar_*); 56.27 (-O-*C*H_3_); 18.52 (=C-*C*H_3_). ATR-FTIR (cm^–1^): 2840 and 2747 (υ_C-H sp3_); 1730 and 1691 (υ_C=O_); 1635 (υ_C=C_); 1593, 1503 and 1456 (υ_C=C aromatic_); 1202 (υ_C-O acyl_); 1120 and 1025 (υ_C-O alkoxy_); 863 and 814 (δ_C-H aromatic_).

### 2.3. Preparation of Monoporous VMA-Based Materials

The monoporous material was prepared through a photochemically-driven free-radical polymerization reaction of the following mixture: VMA as a bio-based monomer, EGDMA as the crosslinker, a porogenic solvent, and DMPA as the initiator (2 wt.% with respect to the total comonomer amount), in a procedure adapted from previous work [17]. The VMA/EGDMA molar ratios of co-monomers (95/5, 90/10, 80/20, 70/30, and 60/40 mol.%) and co-monomer/solvent volume ratios (40/60, 30/70, 25/75, 20/80 and 10/90 vol.%) were varied. In order to determine the optimal conditions, a variety of porogenic solvents, i.e., MeOH, EtOH, *i*-PrOH, *t*-BuOH, 1-octanol, DOH, EtOAc, toluene, and cyclohexane, were used. The polymerization mixtures were homogenized and were transferred into cylindrical molds. The latter were placed in a spectrolinker XL-1500 UV oven (Spectronics, Westbury, NY, USA) equipped with six lamps (6 × 15 W) for 4 h under irradiation at 365 nm so as to trigger the photo-induced free-radical copolymerization of co-monomers. The porogen extraction was performed by evaporating the solvent under reduced pressure for 24 h at room temperature. The monoporous materials were obtained with nearly quantitative yields (95–100%).

### 2.4. Preparation of Monoporous HEMA-Based Materials

HEMA-based materials were prepared according to the same procedure described for the synthesis of monoporous VMA-based materials. HEMA was used as the functional monomer, *i*-PrOH as the porogenic solvent, and DMPA as the initiator (2 wt.% with respect of the total amount of monomers). In addition, the HEMA/EGDMA molar ratio of co-monomers was fixed at 70/30 mol.% and the co-monomer/solvent volume ratio at 20/80 vol.%. Herein, such HEMA-based networks were used as reference systems.

### 2.5. Preparation of Biporous HEMA- Or VMA-Based Materials

To form large oriented tubular pores, nylon yarns of varying diameters were used, i.e., 50, 120, and 400 µm. The latter were held by a cylindrical mold whose two extremities were created by 3-D printing. This system made it possible to maintain the nylon threads and to play on the number of threads present in the materials (i.e., 7, 13, or 37). It is noteworthy that the nylon yarns were periodically aligned in the mold. The entire assembly was then dipped in a polystyrene solution (2 g PS in 10 mL THF) to form a polymeric layer around the nylon yarns. One such polystyrene layer helped removing the nylon threads after the monolith synthesis. The entire assembly was then placed in another larger mold. It was then filled with one of the polymerization mixtures comprising a porogenic solvent to generate the nanoporosity level. Upon free-radical polymerization and subsequent removal of the PS-coated nylon fibers and of the porogenic solvent by readily pulling on the yarns and successive evaporation of the solvent, respectively, biporous materials with nanopores and aligned tubular macropores were generated (1.2 cm diameter, 1 cm height). The biporous anisotropic materials were obtained with nearly quantitative yields (95–100%).

### 2.6. Instrumentation

^1^H and ^13^C NMR spectra were recorded on a Bruker Avance II 400 NMR spectrometer, using resonance frequencies of 400 and 100 MHz, respectively.

Infrared spectra were recorded using a Bruker Tensor 27 DTGS spectrometer in attenuated total reflection (ATR) mode between 4000 and 450 cm^–1^ with an average of 32 consecutive scans and a resolution of 4 cm^−1^.

Raman spectra were recorded using an XPlora One spectrometer from Horiba Jobin Yvon equipped with a laser emitting at 638 nm. The acquisition time was fixed at 1 min.

The porosity ratios, pore volumes and pore size distributions of the materials were determined by mercury intrusion porosimetry (MIP) using an AutoPore IV 9500 porosimeter from Micromeritics. The determination of the porosity features was based on the Washburn equation between the applied pressure (from 1.03 to 206.8 MPa) and the pore size into which mercury intruded.

Scanning electron microscopy (SEM) analyses were performed with a MERLIN microscope from Zeiss equipped with InLens, BSE and SE2 detectors using a low accelerating tension (10 kV) with a diaphragm aperture of 30 μm. The samples were first cryofractured and coated with a 4-nm layer of palladium/platinum alloy in a Cressington 208 HR sputter-coater.

X-ray microtomography experiments were performed using an Ultratom microtomography system manufactured by RX Solutions that combined a 300 mm internal diameter hollow rotation plate, a Hamamatsu L10801 X-ray source (230 kV, 200 W, 5 μm) and two “interchangeable” imaging devices, namely a 2520 V Paxscan Varian flat-panel sensor (1920 × 1560 pixels, pixel size: 127 μm) and an HD PhotonicScience VHR camera (4008 × 2672 pixels, pixel size: 9 µm). The 3D images were reconstructed from the radiographic projections using the X-act software provided by the manufacturer. All images presented in this study were analyzed with Fiji^®^ Freeware.

The measurements of static water contact angles were carried out at room temperature with a Krüss goniometer in the sessile drop configuration. A droplet (5 μL) of Milli-Q water was deposited onto the surface and the recorded image was analyzed by the Drop Shape Analysis software to determine the contact angle value. An average of ten measurements was performed on each film surface.

## 3. Results and Discussion

Porous polymers have been the subject of intense research in the last decades, notably because of their plethora of potential applications in different fields, such as separation sciences [18,19], heterogeneous supported catalysis [20], (nano)filtration techniques [21,22], to cite but a few. In the prospect of achieving model wood-mimicking materials with double porosity, vanillin methacrylate (VMA) was used as a reference monomer. Indeed, the latter was obtained from vanillin which is known to be an aromatic compound widely employed as flavors in foods, fragrances, and pharmaceuticals [23,24]. Vanillin can be produced in two different ways, i.e., either from petroleum-derived phenol, or directly from lignin, which is the most abundant aromatic polymer in nature. Thus, vanillin and its derivatives are considered as attractive bio-based monomers that can mimic the chemical composition of wood. Other building blocks isolated from lignin degradation consist of aromatic molecules bearing hydroxyl and alkoxy groups, e.g., guaiacol and syringol [6,7]. Several applications from vanillin-based materials can be found in the literature. For instance, Peng et al. have developed vanillin cross-linked chitosan microspheres as drug-delivery carriers for the controlled release of resveratrol, a promising drug that has notably advantageous effects against severe diseases, such as cancers [25]. Stanzione et al. have also prepared methacryloyl lignin model compounds, like VMA, to replace styrene in liquid-molding vinyl ester resins. Unfortunately, this monomer could not be used as a viable reactive diluent as it is solid at room temperature [16]. In addition, different reports from Epps’s research group also demonstrated that homopolymers arising from such bio-based compounds (i.e., guaiacol, syringol, and vanillin) could be prepared from corresponding methacrylic monomers and displayed rather similar physicochemical properties than those of polystyrene [6,7].

Previous investigations on the rational design, preparation, characterization and fluid flow transfer properties of poly(2-hydroxyethyl methacrylate) (PHEMA)-based materials with double porosity have been reported by our group and involved different synthetic strategies [17,26,27,28,29,30,31,32]. The double porogen templating approach was adopted by using a porogenic solvent as a nanoporogen and (non-)sintered sieved NaCl particles or poly(methyl methacrylate) (PMMA) beads as macroporogen templates [17,26,27,30]. A complete investigation on the nanoporous network was carried out [17]. The smaller level of porosity was generated through a phase separation process induced during free-radical polymerization. In addition, different parameters could influence the nanoporosity, such as the polarity of the porogenic solvent and its proportion with respect to the co-monomers’ total volume of polymer networks. To obtain the macroporosity, the macroporogen was incorporated into the comonomer/solvent mixture before polymerization and was removed by simple extraction with a suitable solvent. Thus, the macroporosity features depended on the particle shape and on their packing.

**Figure 1:**
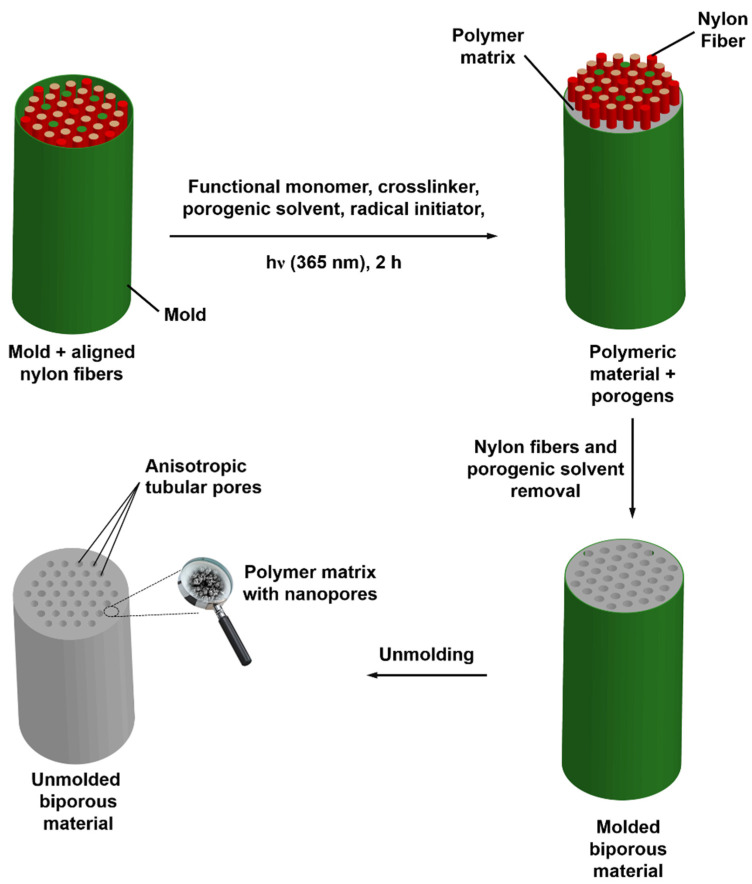
Schematic representation of the general approach implemented for the design of biporous materials prepared from VMA bio-based monomer. The scheme shows here the preparation of a biporous material with 37 anisotropic tubular pores.

In order to mimic the wood structure, the same approach was applied to prepare anisotropic biporous materials. In this work, polymer threads were used as micrometer-sized macroporogens to generate the wood-mimicking vessels, and all the yarns were aligned in the same parallel direction using polymer templates engineered by 3-D printing (Figure 1). A mold specifically designed with such yarn patterns was filled up with the reaction mixture, and they were removed after polymerization. The main characteristics of the larger porosity level depended on the density of polymer threads and on their diameter. Consequently, the novelty of the present approach lay in the development of tailor-made biporous bioinspired materials, which intended to reproduce several complex aspects of wood structure at the origin of several physical processes. Our work does not pretend that the produced materials have better performance than others already published in this research field. It just allows for the preparation of porous frameworks that have a greater resemblance to woodlike structure, even though it is not a perfect replica.

Before the preparation of biporous VMA-containing materials, studies on the formation of small pores were carried out. As the two levels of porosity could be obtained independently, the porosity and the pore size of the nanoporous network could be determined by preparing monoporous materials. The effects of porogenic solvent polarity and co-monomers/solvent volume ratio on these materials were investigated. Once the most appropriate synthetic conditions were found, doubly porous materials were designed using yarns of varying diameters (50, 120, and 400 µm). To prove the formation of the two levels of porosity in a controlled and independent manner, all experiments on these materials were then characterized by SEM and X-ray microtomography.

### 3.1. Effect of Porogenic Solvent Nature on Porous Features of Vanillin-Based Polymeric Materials

**Figure 2:**
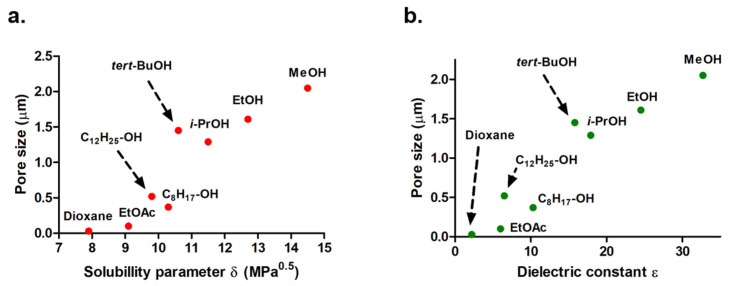
Evolution of pore size in the prepared monoporous materials as a function of (**a**) solubility parameter δ or (**b**) dielectric constant of the porogenic solvent used.

To obtain porous structures, the amounts of porogenic solvent and cross-linking agent should exceed specific threshold values [33]. It is indeed well-known that the nature of the porogenic solvent and its proportion in the polymerization mixture are key parameters which greatly influence the pore size distribution in porous networks [17]. In this context, several porogenic solvents were used to investigate the effects of the polarity, the solubility parameter, and the dielectric constant on the porous features of the resulting polymeric materials, namely methanol (MeOH), ethanol (EtOH), isopropanol (*i*-PrOH), *tert*-butanol (*t*-BuOH), octanol, dodecanol, ethyl acetate (EtOAc), and dioxane. These different solvents were chosen according to their miscibility with the co-monomer mixture before polymerization. The formation of (nano)porous networks was achieved through a phase separation process after complete removal of solvents after polymerization. Porous features obtained by MIP of the VMA-based polymeric materials are gathered in Table 1 while the evolution of the pore size vs. solubility parameter δ and dielectric constant ε is given in Figure 2. It could immediately be noticed that the average pore size of such materials that varied from 100 nm (EtOAc, δ = 9.1 MPa^0.5^ and ε = 6.0) to 2.05 µm (MeOH, δ = 14.5 MPa^0.5^ and ε = 32.7) increased along with the solubility parameter δ and with the dielectric constant ε of the considered porogenic solvent used (Figure 2a,b). From this observation, it could be easily concluded that the more hydrophobic the porogenic solvent (the lower the δ and ε values), the lower the pore size. Only two solvents do not respect this trend: *t*-BuOH and dodecanol. This can be easily explained by the viscosity and the low boiling point of these solvents. With those solvents, higher pores than expected are obtained, that is to say the solvent is expelled faster during the copolymerization, likely due to their physicochemical characteristics.

**Figure 3:**
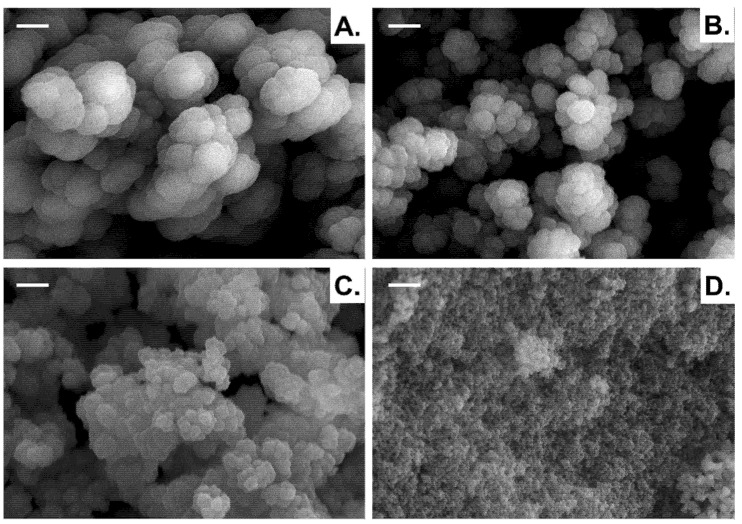
SEM micrographs of monoporous poly(VMA-*co*-EGDMA) materials prepared from 70/30 mol.% VMA/EGDMA and 80 vol.% MeOH (**A**), EtOH (**B**), *i*-PrOH (**C**) or EtOAc (**D**) with respect to the total volume of co-monomers. Scale bar = 1 µm.

In parallel, observation of the porous features of these materials was conducted by SEM, as shown from Figure 3. SEM results were in good agreement with those determined from MIP. Indeed, smaller pore sizes were obtained with octanol as a porogenic solvent. The opposite phenomenon was observed with porous materials based on HEMA since the smallest pore sizes were generated in the presence of a polar solvent [17]. In one such case, an average pore size could be identified from 100 nm to 7 µm. Using nonpolar porogenic solvents, such as octanol or EtOAc, resulted in a unimodal distribution curve, unlike other solvents. When octanol was used as a porogenic solvent, a porous network with pore sizes ranging from 20 nm up to 4 µm, centered at 374 nm was obtained, while a distribution ranging from 10 nm to 1 µm, centered at 98 nm, was obtained when using EtOAc. Besides, the porosity ratio determined by MIP was slightly higher with octanol than with EtOAc (*P*_octanol_ = 71% (δ = 10.3 MPa^0.5^ and ε = 10.3) > *P*_EtOAc_ = 63% (δ = 9.1 MPa^0.5^ and ε = 6.0)). These values were slightly lower compared to the volume of porogenic solvent initially introduced into the polymerization mixture (80 vol.%). This could be explained by a certain non-interconnected nanometric porosity within the polymer material or a lack of phase separation due to the solvent nature [17]. Unlike materials prepared with EtOAc as a porogenic solvent, those developed with octanol required an additional step before drying at room temperature under vacuum, i.e., extraction with dichloromethane. The elaboration of porous materials with a polar solvent, like methanol (δ = 14.5 MPa^0.5^ and ε = 32.7) or ethanol (δ = 12.7 MPa^0.5^ and ε = 24.5), gave rise to rather large pore size distribution curves centered at 2.05 µm and 1.61 µm, respectively. Again, the porosity ratios were rather low compared to the 80 vol.% of solvent in the polymerization mixture. Due to the difference of chemical structures of these monomers when compared to vanillin methacrylate, different trends in terms of porous profiles and porosity ratios were observed in the latter cases.

### 3.2. Effect of Co-Monomers/Solvent Volume Ratio on Nanoporous Vanillin-Based Materials

The effect of the porogenic solvent proportion on the VMA/EGDMA nanoporous materials was investigated by varying the volume percentage of EtOH in the polymerization mixture. EtOH was chosen as a reference porogenic solvent. To this purpose, the polymerization mixture was constituted of 70/30 mol.% of VMA/EGDMA, AIBN (2 wt.% with respect to co-monomers amount), and EtOH. While maintaining constant the molar proportions of the co-monomers and the mass content of AIBN, the final co-monomers/EtOH volume ratio was varied from 5/95 to 35/65 vol.%. MIP measurements were carried out to examine the effect of the solvent proportion on the porosity of the resulting polymeric frameworks. An increase in the pore volume with the co-monomers to solvent volume ratio (from 37% to 86%) was noticed. The higher the amount of porogenic solvent, the smaller the pore sizes. For instance, at 75 and 90 vol.% EtOH, larger macropores were observed centered on 2.27 µm and 1.29 µm, respectively. As expected, the porosity ratio of VMA-based materials decreased when decreasing the volume of porogenic solvent in the polymerization feed (Table 2). Again, the porosity ratios were lower than the initial volumes of EtOH incorporated, since their values were equal to 86%, 71%, and 64% for 95%, 90% and 80%, respectively, still demonstrating a good interconnectivity in the polymeric networks. As shown in Table 2, the total pore volumes of the porous materials were also determined by MIP. Therefore, the amount of porogenic solvent influenced the total pore volume since the latter clearly increased with the amount of porogenic solvent.

### 3.3. Design of Doubly Porous Materials with Varying Macroporogen Sizes and Patterns

**Figure 4:**
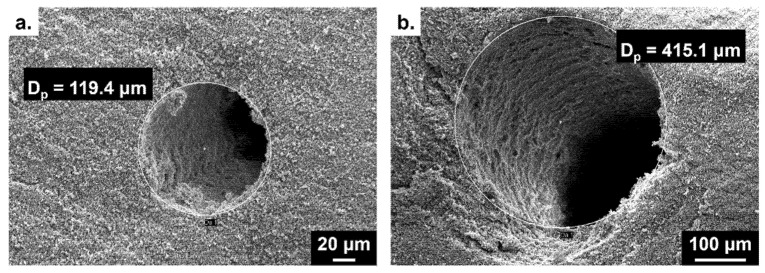
SEM observation of tubular pores obtained in VMA-based materials from nylon fibers with diameters of 120 µm (**a**) and 400 µm (**b**).

**Figure 5:**
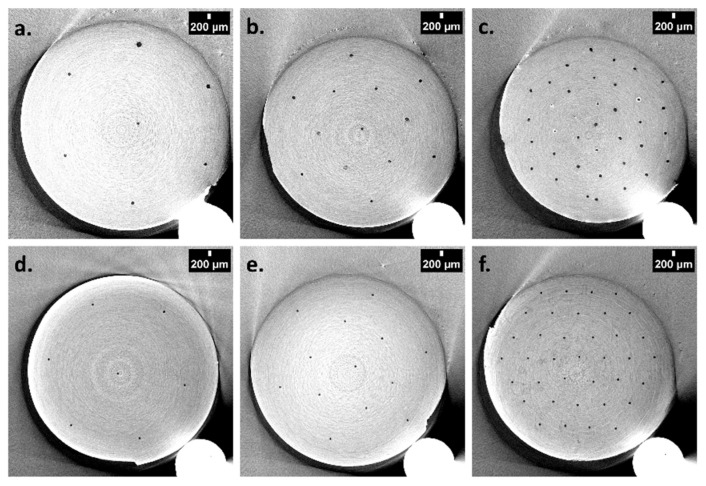
Tomographic images of oriented 120 µm-sized tubular macropores for 7 (**a**), 13 (**b**), and 37 (**c**) holes in HEMA-based materials and oriented 120 µm-sized tubular macropores for 7 (**d**), 13 (**e**), and 37 (**f**) holes in VMA-based materials.

In the present study, ethyl acetate (EtOAc) was selected as the porogenic solvent to produce the lower porosity level as it allowed for an average pore size close or even lower than 100 nm in order to mimic the wood microstructure. Nylon threads with three different diameters, i.e., 50, 120, and 400 µm, were used to generate the second porosity level, which was labeled macroporosity. As schematized in Figure 1, cylindrical molds were used to obtain the polymeric materials with double porosity. In these molds, a mixture of VMA and EGDMA were copolymerized via DMPA-photoinitiated free-radical polymerization in the presence of two porogens, namely EtOAc as a nanoporogen and nylon threads as macroporogens. In parallel to the variation of diameter associated with nylon yarns, another investigation on the variation of the amount of these yarns within the mold was also carried out. To this purpose, three values of 7, 13, and 37 yarns were selected. In Figure 1, the seven-yarn pattern corresponds to the red threads, the 13-yarn pattern to the red and green threads, while the 37-yarn pattern is the total of the red, green and ochre threads. After extraction of the porogenic solvent and nylon yarns and demolding, mercury intrusion porosimetry measurements, scanning electron microscopy (Figure 4) and X-ray microtomography (Figure 5) analyses were performed.

**Figure 6:**
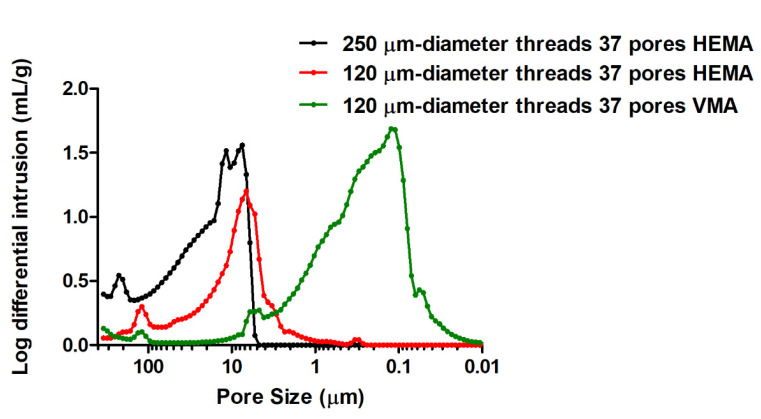
MIP profile of HEMA or VMA-based biporous materials with tubular macropores (thread diameters used: 120 and 250 µm). The polymerization mixture was constituted of 70/30 mol.% HEMA or VMA/EGDMA and 80 vol.% *i*-PrOH or EtOAc with respect to the total volume of comonomers.

In addition, biporous materials based on HEMA were also prepared as reference systems. Mercury intrusion porosimetry profiles of the polymers obtained from HEMA as the functional monomer, *i*-PrOH as the porogenic solvent and 120 or 250-μm nylon yarns demonstrated, as expected, the presence of two porosity levels (Figure 6). The first one, centered around 7 μm, arose from the porogenic solvent upon syneresis during the polymer network formation, while the second one, centered on 120 or 250 μm, was obtained after extraction of the two types of nylon fibers. The MIP results obtained with VMA monomer, EtOAc as the porogenic solvent and 120 μm nylon fibers also showed such a double porosity feature but with a weaker peak corresponding to the tubular macropores arising from nylon yarns. Secondly, the SEM observations permitted to clearly visualize the three-yarn densities (data not shown). It was also possible to verify the diameter of the tubular anisotropic channels present in the resulting materials (Figure 4).

Regardless of the monomer used (HEMA or VMA), the macropore pattern and diameter values arising from nylon fibers were in good agreement with those expected from the macroporogen pattern and size. Indeed, it was noticed that the pore diameter observed was close to or even identical to that of the expected 50, 120, and 400 µm-diameter nylon yarns. Interestingly, the macropores in VMA-based materials were perfectly cylindrical, while HEMA-based materials were constituted of deformed tubular channels (data not shown). On the other hand, X-ray microtomography can be considered as a complementary imaging technique of choice to SEM [32]. This technique permitted to produce fully 3-D images of the whole volume of the sample (internal and external structure). In Figure 5, the three thread densities studied were perfectly visible, which allowed one to ensure the proper arrangement of the yarns in the mold, thus enabling the generation of desired tubular pore pattern within the polymeric scaffold. This technique also confirmed the presence of tubular pores continuously throughout the entire length of the material, as it could be seen from 3-D reconstruction of the pores (data not shown).

From the tomographic observations processed by *Image J^®^*, it was again possible to verify that the macropore diameters were in good agreement with those of the threads used (Table 3). Only the arrangement of the threads within the materials changed from one material to another one, while the diameter of the threads was kept constant at 120 µm. Thus, 50 macropore diameters were precisely measured for each material (*i.e*., 5 macropores per slice) and an average value was thus calculated. In the case of VMA-based materials, the macropore diameters (120.7 µm) were nearly identical to the expected values, while the measured values (168.6 µm) were higher than the expected ones for HEMA-based materials. Such differences could be explained by different stiffness behavior associated with the two matrices. HEMA gave indeed much softer materials than those obtained from VMA, and consequently HEMA-based materials had the tendency to somehow expand during the drying process, thus leading to the observation of larger cylindrical pores than those theoretically expected.

### 3.4. Functionalization of Vanillin-Based Doubly Porous Materials

**Figure 7:**
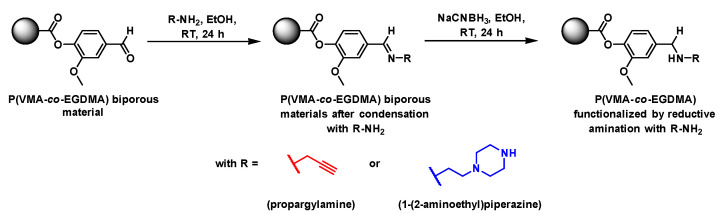
General scheme illustrating the pore surface functionalization of Poly(VMA*-co-*EGDMA) materials by reductive amination with 1-(2-aminoethyl)piperazine or propargylamine.

**Figure 8:**
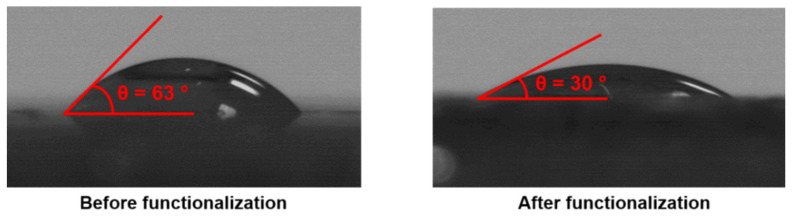
Water contact angle measurements realized on polymers prepared from vanillin methacrylate before and after functionalization with AEP.

The presence of benzaldehyde moieties at the pore surface of VMA-based materials is a real advantage for such bio-based materials as one can envision their further chemical modification through a reductive amination reaction with amino-bearing organic compounds [36,37]. To this purpose, the functionalization of these doubly porous materials was implemented through a two-step synthetic strategy involving the condensation between pending available aldehydes and two different amino compounds, either 1-(2-aminoethyl)piperazine (AEP) or propargylamine (PA), followed by hydride-mediated reduction of the intermediate Schiff base, as depicted in Figure 7. The functionalization of these biporous materials with AEP notably allowed for increasing their hydrophilic character, as shown by water contact angle measurements carried out before and after functionalization from such vanillin-based thin films produced onto glass slides. Indeed, the contact angle decreased on the surface of the VMA-based thin film after functionalization with AEP from 63° to 30°, as the amine functions of AEP increased the hydrophilic nature of the polymeric materials after functionalization. (Figure 8).

**Figure 9:**
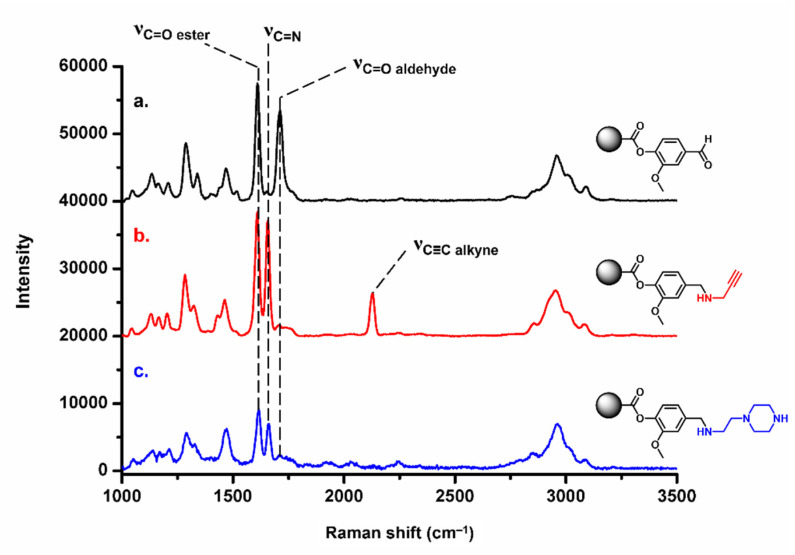
Raman spectra of Poly(VMA*-co-*EGDMA) porous materials before (**a**, black trace) and after reductive amination with propargylamine (**b**, red trace) or 1-(2-aminoethyl)piperazine (**c**, blue trace).

On the other hand, the functionalization of vanillin-based materials with propargylamine permitted to envision further photo-patterned site-specific thiol-yne grafting with other organic molecules. Raman spectroscopy analyses effected before and after functionalization with AEP or PA showed the disappearance of the characteristic signal arising from aldehyde moieties at 1710 cm^–1^, while a new band was immediately noticed at 2130 cm^–1^ after functionalization with PA (Figure 9), attributed to C≡CH stretching vibrations. It is worth mentioning in the Raman spectra of such functionalized porous VMA-based materials the appearance of a band at 1660 cm^–1^. It was attributed to the presence of some nonreduced imine bonds arising from the condensation between available aldehyde functions at the pore surface of the polymers and nucleophilic amines, i.e., AEP and PA.

One such functionalization reaction permitted to tune the materials surface chemistry and potentially played on the interfacial properties of VMA-derived porous polymers in a straightforward fashion.

## 4. Conclusions

This work clearly demonstrated that the design and synthesis of anisotropic bio-based biporous polymeric materials could be achieved via a straightforward and versatile methodologyby combining the double porogen templating approach and 3-D printing. Interestingly, the two porosity levels could be easily tuned by playing with different synthetic parameters. Thus, the nature of the porogenic solvent and its volume fraction in the initial polymerization feed were shown to be crucial parameters to take into account regarding the lower porosity level. On the other hand, the diameter and pattern of nylon yarns used to produce the higher porosity level could be varied to tune the macropore size and periodicity. We notably demonstrated that the pore size of the lower porosity level increased with the polarity of the solvent used, while it decreased with the increase in volume ratio of the porogenic solvent. In parallel, the sizes of the nylon yarns used and their density in the macroporogen template allowed for a fine and independent tuning of the higher porosity level in such anisotropic polymeric monoliths. Finally, we illustrated that vanillin-based materials could constitute a versatile and facile functionalization platform so as to play with the interfacial properties of the resulting bio-based materials. These materials are intended for implementation in fluid flow and transport properties to better understand the physical mechanisms that govern notably the imbibition-drying cycles in wood, a natural building material.

It is worth mentioning that this work could pave the way towards the development of other bio-based biomimetic porous polymeric frameworks, with e.g., guaiacyl or syringyl methacrylate monomers.

## Figures and Tables

**Table 1:** Porous features of monoporous VMA-based networks synthesized using various solvents as porogenic agents. The VMA/EGDMA molar ratio was fixed at 70/30 mol.% and the volume ratio of co-monomers/solvent was equal to 20/80 vol.%.

Solvent	Porosity Ratio ^1^ (%)	Total Pore Volume ^1^ (mL.g^−1^)	Peak Pore Size ^1^ (µm)	Δ ^2^ (MPa^0.5^)	E ^3^
MeOH	69	2.35	2.05	14.5	32.7
EtOH	64	2.50	1.61	12.7	24.5
*i*-PrOH	67	2.59	1.29	11.5	17.9
*t*-BuOH	71	2.78	1.45	10.6	15.8
Octanol	71	1.70	0.37	10.3	10.3
Dodecanol	67	1.46	0.52	9.8	6.5
EtOAc	63	1.30	0.10	9.1	6.0
Dioxane	49	0.69	0.03	7.9	2.2

^1^ Values as determined by mercury intrusion porosimetry. ^2^ Solubility parameters (δ) cited from literature [34]. ^3^ Dielectric constant (ε) cited from literature [35].

**Table 2:** Porous features of monoporous VMA-based networks synthesized using various volume proportions of EtOH as the porogenic solvent.

Vol.% Comonomers (VMA/EGDMA)	Vol.% EtOH	Porosity Ratio ^1^ (%)	Total Pore Volume ^1^ (mL.g^−1^)	Peak Pore Size ^1^ (µm)
5	95	86	3.17	1.29
10	90	71	2.50	1.29
20	80	64	2.30	1.61
25	75	57	2.10	2.27
30	70	48	1.56	6.04
35	65	37	0.85	6.67

^1^ Values as determined by mercury intrusion porosimetry.

**Table 3:** Determination of average macropore diameter using *Image J^®^* software (with a fixed nylon thread diameter of 120 µm).

Number of Threads	Macropore Diameter ^1^ (µm)	
	VMA-Based Materials	HEMA-Based Materials
7	119.3	170.9
13	120.8	166.2
37	122.0	168.8
Mean value	120.7	168.6

^1^ For each material, average values have been calculated from the diameter measurement of 50 distinct macropores.

## Data Availability

Data sharing not applicable.
